# New insight of metabolomics in ocular diseases in the context of 3P medicine

**DOI:** 10.1007/s13167-023-00313-9

**Published:** 2023-02-17

**Authors:** Quyan Zhang, Nan Wang, Yuhua Rui, Yang Xia, Siqi Xiong, Xiaobo Xia

**Affiliations:** 1 Eye Center of Xiangya Hospital, Central South University, 87 Xiangya Road, Changsha, 410008 Hunan Province China; 2 Hunan Key Laboratory of Ophthalmology, Central South University, Changsha, 410008 China; 3 National Clinical Research Center for Geriatric Disorders, Xiangya Hospital, Central South University, Changsha, 410008 China; 4 International Joint Research Center for Medical Metabolomics and Department of Otolaryngology Head and Neck Surgery, Xiangya Hospital, Central South University, Changsha, 410008 China

**Keywords:** Metabolomics, Biomarkers, Ocular diseases, Diabetic retinopathy, Age-related macular degeneration, Glaucoma, Predictive preventive personalized medicine (3PM / PPPM)

## Abstract

Metabolomics refers to the high-through untargeted or targeted screening of metabolites in biofluids, cells, and tissues. Metabolome reflects the functional states of cells and organs of an individual, influenced by genes, RNA, proteins, and environment. Metabolomic analyses help to understand the interaction between metabolism and phenotype and reveal biomarkers for diseases. Advanced ocular diseases can lead to vision loss and blindness, reducing patients’ quality of life and aggravating socio-economic burden. Contextually, the transition from reactive medicine to the predictive, preventive, and personalized (PPPM / 3P) medicine is needed. Clinicians and researchers dedicate a lot of efforts to explore effective ways for disease prevention, biomarkers for disease prediction, and personalized treatments, by taking advantages of metabolomics. In this way, metabolomics has great clinical utility in the primary and secondary care. In this review, we summarized much progress achieved by applying metabolomics to ocular diseases and pointed out potential biomarkers and metabolic pathways involved to promote 3P medicine approach in healthcare.

## Introduction

Metabolomics is the profiling of metabolites in biofluids, cells, and tissues [1]. The metabolome comprises a myriad of small molecules below 1.5 kDa, including amino acids, carbohydrates, nucleosides/nucleotides, tricarboxylic acid intermediates, and lipids. Metabolites are the downstream of genes, ribonucleic acid (RNA), proteins, and its interactions with environment, reflecting the real functional states of an individual. Not only does metabolomics give expression to the influences of genomics, transcriptomics, proteomics, and environment, but also interacts with other omics [2]. Metabolomics has been applied to identify biomarkers for the early diagnosis and prediction of diseases and explore potential preventive and therapeutic targets [3–7].

There are two tools applied in metabolomics profiling: nuclear magnetic resonance (NMR) spectroscopy and mass spectrometry (MS). NMR spectroscopy is the detection of radiofrequency signals released by the energetic transition of nuclei of ^1^H in a strong magnetic field. By analyzing the frequency spectrum, various metabolites can be identified, and that is how researchers get the metabolome of a certain bio-sample. There are several advantages of NMR. The principle of NMR decides the noninvasiveness of this technic, enabling the sample to be used for other studies. Also, it requires little sample preparation. The results of NMR are quantitative, with robustness and accuracy. The reproducibility of NMR measurements enables the comparison between different labs. The limitation of NMR is lack of sensitivity, as NMR only detects the most abundant metabolites (≥ 1 μM) [8], and the range of metabolites of NMR is not as wide as that of MS.

MS has become the most popular analytical platform in metabolomics, due to its sensitivity and large scale of phenotyping [9]. It is a technique to identify the components of a sample through their mass and electrical charge. In a mass spectrometer, the procedure includes four steps: ionization, acceleration, deflection, and detection, and then a mass spectrum with the mass-to-charge (m/z) ratio of components in the sample is depicted. MS is always coupled to different separation technologies, including liquid chromatography (LC), capillary electrophoresis (CE), and gas chromatography (GC). After the multiple components in mixtures are separated through LC or GC, the effluent is directed to the mass spectrometer to identify each separated component [10]. In the field of metabolomics, LC is the most widely used technology, with its wide range of metabolites and elimination of chemical derivatization [11].

Metabolomics can be classified into targeted and untargeted metabolomics. Targeted metabolomics is a quantitative analysis, revealing the quantity of a specific metabolite, while untargeted metabolomics discloses the entire set of metabolites contained in the sample.

**Fig. 1:**
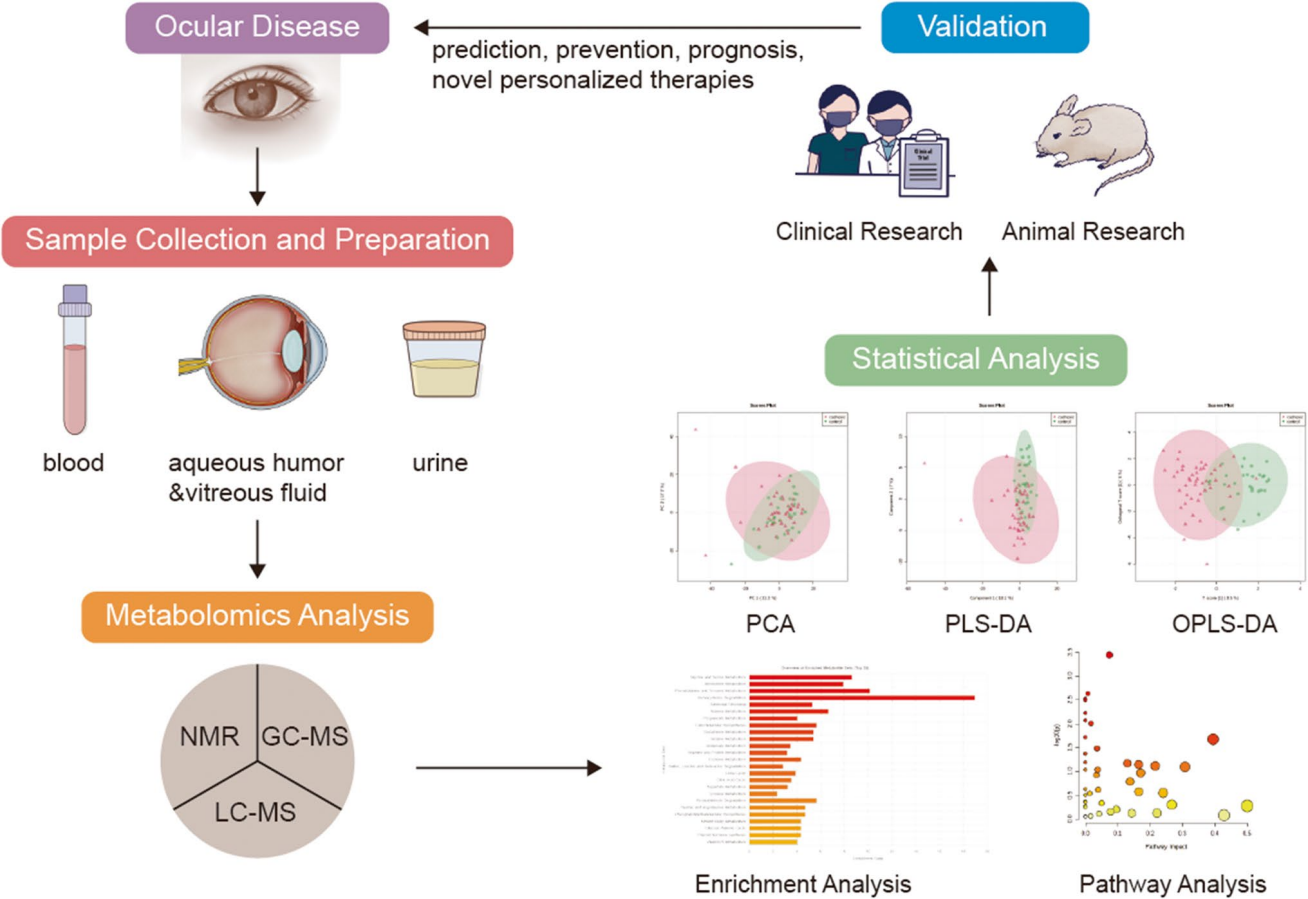
The procedure of metabolomics. The first step is to find a target ocular disease and design the experiment. Next, collect the biofluids of patients, and prepare samples according to the protocol. The third step is metabolomics analysis via NMR, GC–MS, or LC–MS. The fourth step is to analyze the metabolomics raw data for metabolite identification and metabolite quantification. With the information of identified metabolites, bioinformatic analysis is used to assess the metabolic pathways that are related to the phenotype. Principal component analysis (PCA) is the most commonly used unsupervised technique to find the components that best explain the variance in the dataset. Partial least squares-discriminant analysis (PLS-DA) is a commonly used supervised technique to analyze the capability of components to differentiate the different groups. Receiving operator characteristic (ROC) curve analysis is used to evaluate the diagnostic value of a certain metabolite. Enrichment analysis or pathway analysis shows the differential pathways. Once a group of metabolites is identified, the fifth step is to validate it in human validation set or on animal models. The relevant disturbed metabolomic pathways and potential biomarkers may help the prediction, prevention, prognosis of ocular diseases, and the discovery of novel personalized therapies

The typical workflow of untargeted metabolomics includes sample collection and preparation, metabolomics analysis, statistical analysis, and biomarker validation (Fig. 1). In the field of ophthalmology, metabolomics has been widely used [12]. The commonly analyzed biological samples include blood, tear, aqueous humor (AH), and vitreous fluid. Sample collection and processing have been illustrated in detail in previous review [13]. Serum and plasma are the most commonly used biofluids. However, due to the existence of blood–aqueous and blood–retinal barriers, the eye has its own distinct metabolome, so the metabolome in blood may have limitations in reflecting metabolic state in eyes [12]. AH and vitreous fluid are better representative for local metabolism. AH is a transparent fluid in the anterior chamber of the eye, providing nutrition for avascular tissues and discharging metabolic wastes to the venous blood. Vitreous fluid is a transparent fluid between the lens and the retina of the eye. Both AH and vitreous fluid are crucial for stability of intraocular metabolism. The alterations in metabolites of these biological fluids reflect physiological or pathophysiological changes. There is no denying that the metabolome has great potential in the prediction and early diagnosis of ocular diseases and the development of preventive strategies and therapeutic targets. Moreover, the stratification of patients into subgroups based on molecular biomarkers paves way for personalized medicine. Therefore, further development of metabolomics and its application in ocular diseases contributes to a more advanced medicine strategy, namely predictive, preventive, and personalized medicine (3PM/PPPM) [14].

Here we summarized the application of metabolomics in ocular diseases. The metabolites relevant to ocular diseases are summarized in Table 1. Also, we propose the existing limitations of metabolomics. This review integrates the published work to date and summarized potential biomarkers or pathways in different studies, thus providing a comprehensive understanding of the potential of metabolomics in ocular diseases in the framework of 3P medicine. We believe that the application of metabolomics in ocular diseases is an effective way to improve personal outcomes and healthcare systems [56].

**Table 1:** Differential metabolites associated with ocular diseases

Disease	Authors	Samples	Platform	Metabolites (increased level)	Metabolites (decreased level)	Reference
PDR	Barba et al	Vitreous fluid	^1^H-NMR	Lactate, glucose, alanine, acetate	Succinate, galactitol, ascorbic acid	[15]
PDR	Haines et al	Vitreous fluid	LC–MS	Proline, citrulline, aspartate, pentose phosphate, pyruvate, guanine, inosine, hypoxanthine, urate, allantoate, octanoylcarnitine, decanoylcarnitine	Ascorbate, 5-oxoproline, fumarate, glyceraldehyde 3-phosphate, 2/3-phosphoglycerate, xanthine, α-ketoglutarate	[16]
PDR	Paris et al	Vitreous fluid	LC–MS/MS	Octanoylcarnitine, decanoylcarnitine, methionine, allantoin, glutamate, lysine, arginine, proline, citrulline, ornithine	N-acetylaspartate, iditol, glycerate, N-acetylglutamate	[17]
PDR	Tomita et al	Vitreous fluid	LC–MS/MS	Glycine, lactate, pyruvate, proline, allantoin, urate, citrulline, ornithine, dimethylglycine, N-acetylserine, α-ketoglutarate	Creatine, succinate	[18]
PDR	Lin et al	Vitreous fluid	LC–MS/MS	5-HETE, 12-HETE, 20-HETE, 20-COOH-AA, 11,12-EET	-	[19]
PDR	Zhao et al	Vitreous fluid	LC–MS/MS	PGJ2, PGF2α, 12-HETE, 14,15-DiHETrE, ARA, DTA, 9-HODE, 9-OxoODE, 13-OxoODE, 9(10)-EpOME, 12(13)-EpOME, 9(10)-DiHOME, 12(13)-DiHOME, 8-HETrE, 15-HETrE, 9-HOTrE, 13-HOTrE, EPA, 19,20-EpDPE, DHA		[20]
DR	Wang et al	Vitreous fluid	GC–MS	Pyruvate, ornithine, uric acid, pyroglutamic acid, creatinine, leucine, alanine, threonine, lysine, valine, phenylalanine, alloisoleucine, glutamine	Myoinositol, hydroxylamine	[21]
DR	Jin et al	Aqueous humor	NMR	Asparagine, histidine, glutamine, threonine, DMA	Lactate, succinate, 2HB	[22]
DR	Wang et al	Aqueous humor	GC–MS	Glucose	D-2,3-dihydroxypropanoic acid, isocitric acid, threonic acid, myoinositol, lactic acid, citrulline, fructose 6-phosphate	[21]
DR	Chen et al	Plasma	GC–MS	1,5-Gluconolactone, 2-deoxyribonic acid, 3,4-DHBA, erythritol, gluconic acid, lactose/cellobiose, maltose/trehalose, mannose, ribose, urea	1,5-Anhydroglucitol	[23]
PDR	Zhu et al	Plasma	LC–MS	Fumaric acid, uridine, acetic acid, cytidine, 3-methylxanthine, sulfate	3-Sulfinoalanine	[24]
NPDR, PDR	Sumarriva et al	Plasma	LC–MS/MS	Arginine, citrulline, glutamic γ-semialdehyde, dehydroxycarnitine	-	[25]
NPDR, PDR	Peters et al	Plasma	LC–MS/MS	Arginine, citrulline	-	[26]
NPDR	Peng et al	Plasma	LC–MS/MS	-	PGF2α	[27]
NPDR, PDR	Curovic et al	Plasma, serum	GC–MS, LC–MS	2,4-DHBA, 3,4-DHBA, ribonic acid, ribitol	TG(50:1), TG(50:2)	[28]
wet AMD	Han et al	Aqueous humor	LC–MS	Citrate, isocitrate, glutamate	Succinate, α-ketoglutarate, glutamine	[29]
wet AMD	Han et al	Aqueous humor	LC–MS/MS	Deoxycarnitine, TML, glycine betaine, itaconic acid, cis-aconitate	L-proline, N-fructosyl isoleucine, creatine, 5-aminopentanoic acid, norleucine, L-phenylalanine, L-carnitine, γ-glutamylglutamine, hetisine, 3-phenyllactic acid, lysoPC(18:2), coumaroyl agmatine, N-acetylhistidine	[30]
AMD	Laíns et al	Plasma	LC–MS/MS	Adenosine	Diacylglycerols, PCs	[31]
AMD	Acar et al	Plasma, serum	NMR	HDL, triglycerides	VLDL, citrate, phenylalanine, alanine, isoleucine, leucine, and tyrosine, remnant C, apoB, MUFA, SFA, total fatty acids	[32]
wet AMD	Mitchell et al	Plasma	LC–MS/MS	9-Hexadecenoyl carnitine, heptadecanoyl carnitine, octadecenylcarnitine, L-palmitoyl carnitine, stearoyl carnitine	-	[33]
wet AMD	Barca et al	Plasma	LC–MS/MS	Valine, lysine, valerylcarnitine, carnitine, proline	Carnosine	[34]
wet AMD	Mitchell et al	Plasma	LC–MS/MS	Heptadecanoyl carnitine, octadecenylcarnitine, linoleyl carnitine, linolenyl carnitine, glutaconylcarnitine, stearoylcarnitine, lysoSM, lysoPC, linoleoyl ethanolamide, N-oleoylethanolamine, 25-hydroxyvitamin D2, cortexolone, pyroglutamic acid	Kynurenine	[35]
AMD	Orban et al	Serum	LC–MS/MS	ARA	DHA	[36]
AMD	Deng et al	Plasma	LC–MS/MS	12-HEPE, 12-HETE, 4-HDHA, 9-HETE, 14-HDHA, 15-oxoETE, 2,4-dihydroxybenzoic acid, 1,2,3-trihydroxybenzene, 1-methyluric acid, trimethylamine N-oxide, L-tryptophanamide, 1-methylxanthine	Carbamoyl phosphate, UDP-glucose	[37]
AMD	Laíns et al	Plasma	LC–MS/MS	TML, methylsuccinate	Phenylalanine, N-methylhydroxyproline, ribitol, N-palmitoyl-sphingosine, pregnenediol disulfate, 1-linoleoyl-2-linolenoyl-GPC	[38]
POAG	Buisset el al	Aqueous humor	LC–MS	Creatinine, carnitine, acetyl-carnitine, propionyl-carnitine, butyryl-carnitine, glutamine, glycine, alanine, leucine, isoleucine, acetyl-ornithine, hydroxyl-proline, PCs, lysoPC, SM	Taurine, spermine	[39]
POAG	Breda et al	Aqueous humor	NMR	Alanine, N-acetylglutamate, lysine, glutamine/glutamate, α-ketoglutarate, creatine/phospho-creatine, creatinine, glucose, taurine, betaine	Valine, β-hydroxybutyrate	[40]
POAG	Tang et al	Aqueous humor	LC–MS/MS	3′-Sialyllactose, dulcitol, lysoPCs, uric acid, phenyllactate, hydroxyphenyllactic acid, barbituric acid, L-3-phenyllactic acid, PAF, N6-succinyl adenosine, D-sorbitol	Cyclic AMP, 2-methylbenzoic acid, hypoxanthine, xanthosine	[41]
POAG	Tang et al	Plasma	LC–MS/MS	D-mannitol, inosine, hypoxanthine, guanidinoethyl sulfonate, hypoxanthine-9-β-D-arabinofuranoside, p-aminobenzoate	3-Propionic acid, N-lactoyl-phenylalanine, 9-hpode, hydroxyacetone, 2-aminoadipic acid	[41]
POAG	Burgess et al	Plasma	LC–MS/MS	Palmitoylcarnitine, sphingosine/sphinganine	Sphingosine-1-phosphate, hydroxyergocalciferol, ergostanol	[42]
POAG	Umeno et al	Serum	LC–MS/MS	9-HODE, 13-HODE, 5-HETE, 12-HETE, 15-HETE	-	[43]
Glaucoma	Javadiyan et al	Serum	LC–MS/MS	ADMA, SDMA	-	[44]
POAG	Nzoughet et al	Plasma	LC–MS	N-acetyl-L-leucine, arginine, RAC-glycerol 1-myristate, 1-oleoyl-RAC-glycerol, cystathionine	Nicotinamide, hypoxanthine, xanthine, 1-methyl-6,7-dihydroxy-1,2,3,4-tetrahydroisoquinoline	[45]
DED	Pieragostino et al	Tear	LC–MS/MS	-	Cortisol, ADIONE, 17-OHP	[46]
High myopia	Barbas-Bernardos et al	Aqueous humor	LC–MS	Aminooctanoic acid, L-arginine, citrulline, butyryl-L-carnitine, pantothenic acid, sphinganine, histidinyl-phenylalanine, PC(O-32:2)//PC(P-32:1), C24 sulfatide, LacCer(d40:0)	Aminocyclohexanecarboxylic acid, aminoundecanoic acid, dodecanedioic acid, trihydroxyphenyl-γ-valerolactone, didehydro-retinoic acid, L-cysteinylglycine disulfide, dihydropteroic acid, dimethylnonanoyl carnitine, PC(42:6), trihexosylceramide (d36:2)	[47]
Myopia	Kearney et al	Serum	LC–MS/MS	Melatonin	Dopamine	[48]
High myopia	Dai et al	Serum	LC–MS	Seryltryptophan, DG(8:0/17:0/0:0), PE(20:3/22:6), lysoPE(22:4/0:0), 25-hydroxyvitamin D2-25-glucuronide,, γ-glutamyltyrosine, 5-hydroxytryptamine	5-Methyltetrahydrofolic acid, 12-oxo-20-trihydroxy-leukotriene B4	[49]
RRD	Li et al	Vitreous fluid	LC–MS	3-Ethylmalate, creatinine, urea, D-glucuronolactone, uric acid, homoisocitrate, cyromazine, 3-methylhistidine, hypoxanthine, citrate, glycerate, allantoate, 2-oxoglutarate, D-glyceraldehyde, tyrosine, succinate, inosine, phenylalanine, linoelaidic acid	4-Oxoproline, L-carnitine, creatine, valine, lactate, threonate, leucine, isoglutamine, 5-hydroxykynurenamine, ascorbate, tryptophan	[50]
KC	Karamichos et al	Tear	LC–MS/MS	Isocitrate, malate, 1,3 diphosphateglycerate, 3-phosphoglycerate, aspartate	Aconitate, ornithine	[51]
KC	DaphneTeh et al	Serum	LC–MS	DHEA-S, eicosanoids	PS(17:2/20:4)	[52]
Cataract	Tsentalovich et al	Lens	NMR, LC–MS	Acetate, formate, glucose	ADP, AMP, NAD, creatine, carnitine, myo-inositol, 3OHKG, AHBG, GSH-3OHKG	[53]
Cataract	Yanshole et al	Lens, aqueous humor	NMR, LC–MS	-	Amino acids, peptides, myo-inositol, hypoxanthine, inosine, ascorbate, ergothioneine, 3OHKG, AHBG, GSH-3OHKG	[54]
MacTel	Bonelli et al	Serum	LC–MS/MS	Fatty acids, phosphatidylethanolamines, glycerolipids, PCs, lysoPC, lysoPE	Glycine, serine, γ-glutamylglycine, α-ketoglutarate, arginine, ornithine, guanidinoacetate, methionine, betaine, SM, choline	[55]

*PDR* proliferative diabetic retinopathy, *NMR* nuclear magnetic resonance, *LC* liquid chromatography, *MS* mass spectrometry, *HETE* hydroxyeicosatetraenoic acid, *20-COOH-AA* 20-carboxy-arachidonic acid, *EET* epoxyeicosatrienoic acid, *PGJ2* prostaglandin J2, *PGF* prostaglandin F, *DiHETrE* dihydroxy-eicosatrienoic acid, *ARA* arachidonic acid, *DTA* docosatetraenoic acid, *HODE* hydroxy-octadecadienoic acid, *OxoODE* Oxo-octadecadienoic acid, *EpOME* epoxy-octadecenoic acid, *DiHOME* dihydroxy-octadecenoic acid, *HETrE* hydroxy-eicosatrienoic acid, *HOTrE* hydroxy-octadecatrienoic acid, *EPA* eicosapentaenoic acid, *EpDPE* epoxy-docosapentaenoic acid, *DHA* docosahexaenoic acid, *DR* diabetic retinopathy, *GC* gas chromatography, *DMA* dimethylamine, *2HB* 2-hydroxybutyrate, *DHBA* dihydroxybutyric acid, *NPDR* non-proliferative diabetic retinopathy, *TG* triglycerides, *AMD* age-related macular degeneration, *TML* N6-trimethyl-L-lysine, *PC* phosphatidylcholine, *HDL* high-density lipoprotein, *VLDL* very low-density lipoprotein, *remnant C* remnant cholesterol, *apoB* apolipoprotein B, *MUFA* monounsaturated fatty acids, *SFA* saturated fatty acids, *HEPE* hydroxy-eicosapentaenoic acid, *HDHA* hydroxy-docosahexaenoic acid, *oxoETE* oxo-eicosatetraenoic acid, *UDP* uridine diphosphate, *POAG* primary open-angle glaucoma, *AMDA* asymmetric dimethylarginine, *SDMA* symmetric dimethylarginine, *DED* dry eye disease, *ADIONE* 4-androstene-3,17-dione, *17-OHP* 17α-hydroxyprogesterone, *LacCer* lactosylceramide, *DG* diacylglycerol, *PE* phosphatidylethanolamine, *RRD* rhegmatogenous retinal detachment, *KC* keratoconus, *DHEA-S* dehydroepiandrosterone sulfate, *PS* phosphatidylserine, *ADP* adenosine diphosphate, *AMP* adenosine monophosphate, *NAD* nicotinamide adenine dinucleotide, *3OHKG* 3-hydroxykynurenine O-b-D-glucoside, *AHBG* 4-(2-amino-3-hydroxyphenyl)-4-oxobutanoic acid O-b-D-glucoside, *GSH-3OHKG* glutathionyl-3-hydroxykynurenine O-b-D-glucoside, *MacTel* macular telangiectasia type 2)

## Application of metabolomics in ocular diseases

### Diabetic retinopathy

**Fig. 2:**
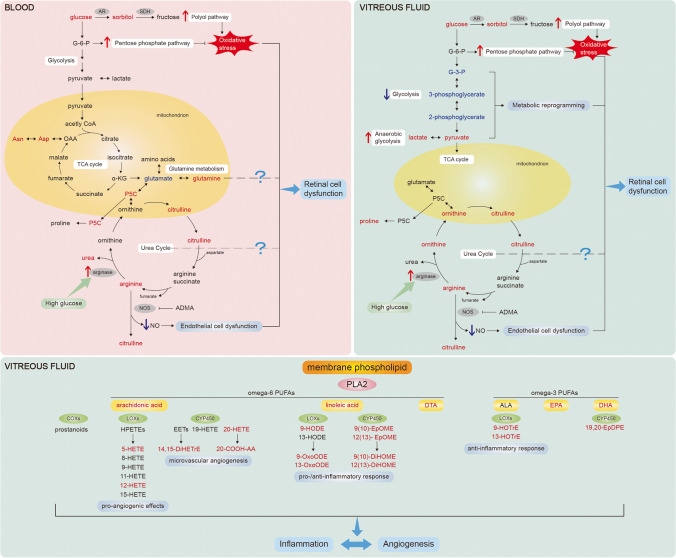
The main disturbed metabolites and metabolic pathways in diabetic retinopathy. Blood and vitreous fluid are the two most common used biofluids in metabolomics of diabetic retinopathy. The mainly disturbed metabolic pathways involved glucose metabolism, polyol pathway, pentose phosphate pathway, urea cycle, arginine metabolism, and lipid metabolism. The red color represents the increased level of metabolites, while the blue color represents the decreased level of metabolites. See text for details. AR, aldose reductase; SDH, sorbitol dehydrogenase; G-6-P, glucose-6-phosphate; Asn, asparagine; Asp, aspartate; OAA, oxaloacetate; TCA cycle, tricarboxylic acid cycle; α-KG, α-ketoglutarate; P5C, pyrroline-5-carboxylate; NOS, nitric oxide synthase; NO, nitric oxide; ADMA, asymmetric dimethylarginine; G-3-P, glyceraldehyde 3-phosphate; PLA2, phospholipase A2; PUFA, polyunsaturated fatty acid; COX, cyclooxygenase; LOX, lipoxygenase; HPETE, hydroperoxyeicosatetraenoic acid; HETE, hydroxyeicosatetraenoic acid; CYP450, cytochrome P450; EET, epoxyeicosatrienoic acid; DiHETrE, dihydroxy-eicosatrienoic acid; 20-COOH-AA, 20-carboxy-arachidonic acid; HODE, hydroxy-octadecadienoic acid; OxoODE, oxo-octadecadienoic acid; EpOME, epoxy-octadecenoic acid; DiHOME, dihydroxy-octadecenoic acid; DTA, docosatetraenoic acid; ALA, α-linolenic acid; HOTrE, hydroxy-octadecatrienoic acid; EPA, eicosapentaenoic acid; DHA, docosahexaenoic acid; EpDPE, epoxy-docosapentaenoic acid

Diabetic retinopathy (DR) is one of the most common microvascular complications of diabetes mellitus [57]. The early stage is termed as non-proliferative diabetic retinopathy (NPDR) [58]. Severe NPDR is more likely to develop into neovascularization, also known as proliferative diabetic retinopathy (PDR). Another pathological change is diabetic macular edema (DME), the fluid accumulation in the macular region. In 2020, it was estimated that more than 150 million persons worldwide had DR, and the number will increase to 233 million by 2045 [59]. It was the fifth cause of blindness and caused 0.86 million cases of blindness in those aged 50 years and older in 2020 [57]. Permanently growing population of patients with DR leads to serious individual health problems and a major public health challenge. Furthermore, DR is a disease with a long preclinical state, whereby the patient is largely asymptomatic, which means that the patient probably cannot be diagnosed until unreversible visual impairment has occurred [60]. Despite the curative effects of anti-vascular endothelial growth factor (anti-VEGF) agent treatment, the effective rate has not reached 50% [61]. Hence, current reactive medicine in diabetic retinopathy is considered being inadequate, and cost-effective medical approaches are needed [62]. It is evident that the paradigm shift from reactive medicine to 3P medicine helps to develop a nationwide screening program, an early in vitro diagnostic, predictive and preventive medical approaches, and promising treatments [14, 63, 64]. It has been proved that when individualized treatment algorithms are applied, long-term outcomes of DR patients can be improved [65, 66]. Only by understanding the molecular and pathophysiological mechanisms of the disease can these advanced healthcare approaches be possible, and metabolomics is one suitable way to reveal the pathophysiological changes (Fig. 2).

Vitreous metabolomics has been thought closer to the metabolic characteristics of retina. Oxidative stress has been proved to be a critical contributor to DR [67]. Barba et al. analyzed the vitreous fluid samples from 22 PDR patients and 22 non-diabetic controls via ^1^H-NMR [15]. Glucose, lactate, and acetate levels were elevated, while galactitol and ascorbic acid levels were reduced. Elevated lactate levels reflected tissue acidosis and anaerobic glycolysis. Notably, decreased galactitol level reflected the activation of the polyol pathway, together with decreased ascorbic acid level, supporting the role of oxidative stress in DR [67, 68]. Haines et al. analyzed the vitreous fluid samples from 9 PDR patients, 25 rhegmatogenous retinal detachment (RRD) patients, and 9 non-diabetic controls via LC–MS [16]. It revealed that downstream metabolites of glycolysis (glyceraldehyde 3-phosphate and 2/3-phosphoglycerate) were decreased, while pentose phosphate pathway product (pentose phosphate) was increased, same to the alteration under oxidative stress [69]. Furthermore, decreased level of xanthine was found to be the strongest predictor of DR, in keeping with the increased level of guanine, inosine, and hypoxanthine (upstream metabolites of xanthine). In this way, the detection of metabolites related to oxidative stress may serve as biomarkers in early prediction and treatment of DR [70].

Arginine metabolism is another disturbed pathway involved in DR. Paris et al. analyzed the vitreous fluid samples from 9 PDR patients with 11 non-diabetic controls via LC–MS/MS [17]. It revealed that arginine metabolism and urea cycle were two of the most perturbed pathways. In retina, arginine can be metabolized in two ways: generating ornithine and urea catalyzed by arginase, and producing nitric oxide (NO) and citrulline catalyzed by nitric oxide synthase (NOS). The overactivity of arginase may lead to the deficiency of arginine in NOS pathway, thus generating superoxide and less NO [71]. This leads to the physiological disturbance of both vascular endothelial and neural cells and the increase of oxidative stress. Moreover, the level of creatine, the downstream of arginine, was decreased in PDR patients, as well as pathways involved with creatine [18]. In oxygen-induced retinopathy (OIR) mouse model, oral administration of creatine suppressed neovascularization. Several studies revealed the therapeutic potential of creatine supplementation on DR, as well as preventive approaches [72].

The role of arachidonic acid (ARA) and other oxylipins in retinal pro-angiogenesis has been concerned in DR [73]. Lin et al. analyzed the vitreous fluid samples from 31 PDR patients and 13 non-diabetic controls via LC–MS/MS [19]. Oxylipins catalyzed by lipoxygenase (LOX) and cytochrome P450 (CYP) were the most affected, with increased levels of 5-HETE (hydroxyeicosatetraenoic acid), 12-HETE, 20-HETE, and 20-COOH-AA (20-carboxy-arachidonic acid). Zhao et al. analyzed the vitreous fluid samples from 41 PDR patients and 22 non-diabetic controls via LC–MS/MS [20], indicating 21 differential oxylipins. In agreement with the previous study, oxylipins involved in LOX and CYP pathways were the most affected. These results reflected the imbalanced inflammation homeostasis in PDR.

There are also few studies on the metabolic composition of AH in DR. Jin et al. analyzed the AH samples from 13 DR and cataract patients, 14 cataract patients, and 7 non-diabetic controls via ^1^H-NMR [22]. This study reported decreased levels of lactate and succinate and increased levels of asparagine, glutamine, histidine, threonine, and dimethylamine (DMA). DMA is a metabolite of asymmetric dimethylarginine (ADMA), contributing to oxidative stress by causing NOS uncoupling. Hence, the authors speculated about the association between increased levels of DMA and oxidative stress and endothelial dysfunction in DR [74]. Wang et al. analyzed the AH samples from 23 PDR patients and 25 non-diabetic controls via GC–MS [21]. Disturbances in ascorbate-aldarate metabolism, glycolysis or gluconeogenesis, galactose metabolism, and arginine and ornithine metabolism were detected in DR. These disturbed metabolism pathways together reflected the oxidative stress, energy metabolism deficiencies, and endothelial dysfunction in DR patients, providing evidences for the pathophysiological process in DR [75, 76].

Blood samples are also crucial sources. In line with the study of vitreous humor [16], pentose phosphate pathway was identified as key metabolic dysregulation associated with oxidative stress in DR [23, 24]. In addition, the role of arginine metabolism in DR is complicated. In accordance with the increased levels of arginine and citrulline in vitreous humor, arginine and citrulline were elevated in the plasma of DR patients [25]. However, the levels of ornithine or proline did not increase correspondingly [26]. Altered lipid metabolism was also detected in the blood samples of DR. Peng et al. analyzed plasma samples from 28 NPDR patients, 22 diabetic controls, and 16 non-diabetic controls via LC–MS/MS [27]. The analysis showed that prostaglandin F2α (PGF2α) was the metabolite that is most negatively relevant to the incidence of NPDR. Furthermore, in vitro study proved that PGF2α increased retinal pericyte mobility via prostaglandin F receptor (FP receptor), and in vivo study proved that latanoprost (FP receptor analogue) partially reversed the reduction of retina capillary. Therefore, PGF2α was identified as a protective metabolite for NPDR and may service as a predictive biomarker for the incidence of NPDR. Rhee et al. analyzed plasma samples from 124 DR patients, 59 diabetic controls, and 32 non-diabetic controls via GC–MS and LC–MS, and glutamine and glutamate were identified as biomarkers for the prediction and prognosis of DR [77]. Also, Chen et al. analyzed plasma samples from 40 DR patients and 40 non-diabetic retinopathy (NDR) controls via GC–MS [23]. They found that 1,5-gluconolactone, 2-deoxyribonic acid, gluconic acid, and urea could be metabolite markers for DR and improved existing risk stratification after adjustment for established risk factors.

Moreover, metabolomics is applied as risk stratification of DR. This first large cohort study on type 1 diabetes mellitus (T1DM) individuals further manifested the disturbances regarding glucose and lipid metabolism [28]. Curovic et al. analyzed plasma and serum samples from 141 T1DM patients with NDR, 90 mild NPDR patients, 186 moderate NPDR patients, 121 PDR patients, 107 PDR patients with fibrosis, and 16 health cases via GC–MS and LC–MS [28]. Four metabolites (2,4-DHBA, ribonic acid, ribitol, and 3,4-DHBA) were positively related to DR stages. 3,4-DHBA was identified as an independent risk marker for progression in DR stage, not only the biomarker for DR [23].

In conclusion, metabolomics can identify the disturbed metabolites in DR, biomarkers for the prediction, prognosis and stratification of DR, and potential therapeutic targeted metabolites for the prevention and personalized treatments of DR [78]. By utilizing metabolomics, the paradigm shift to 3P medicine is cost-effective for both patients and health systems [64].

### Age-related macular degeneration

**Fig. 3:**
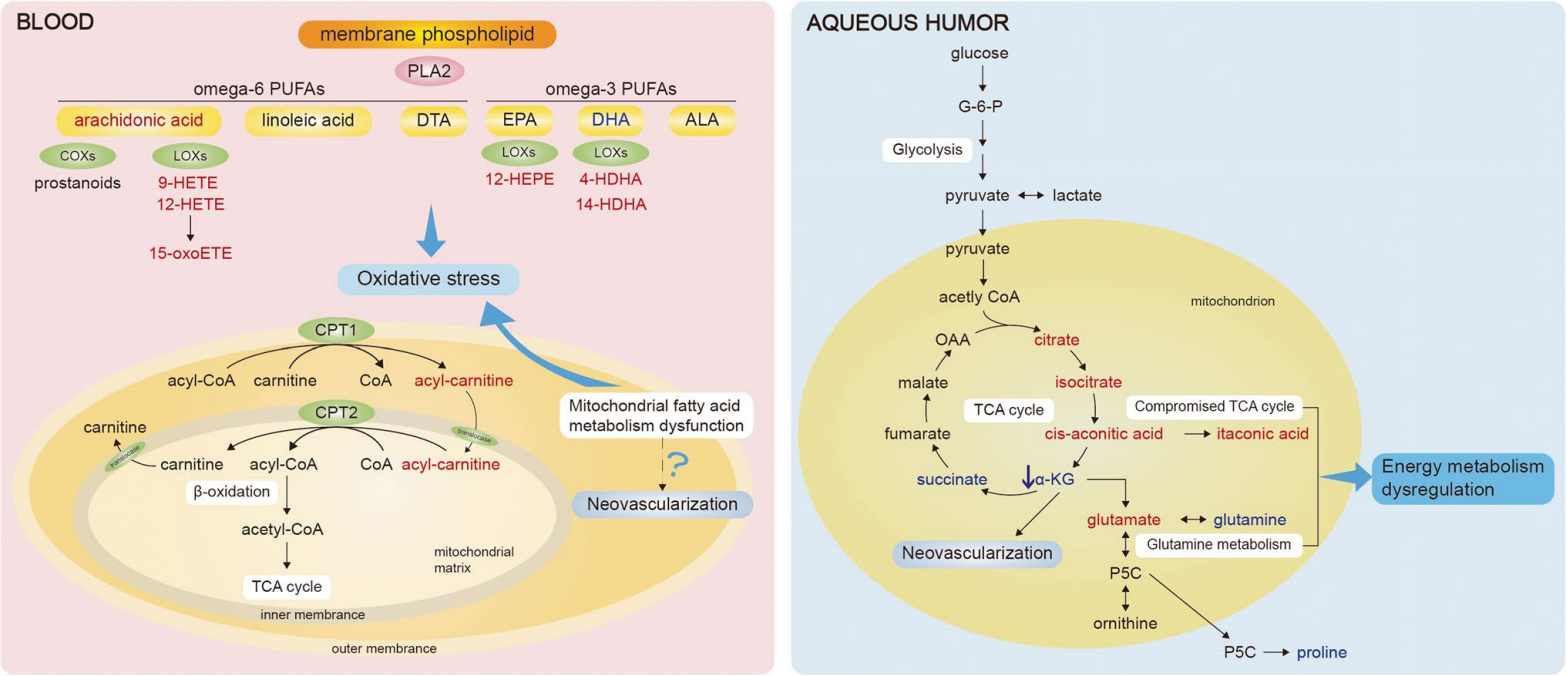
The main disturbed metabolites and metabolic pathways in age-related macular degeneration. Blood, vitreous fluid, and aqueous humor samples are analyzed in metabolomics of age-related macular degeneration. The mainly disturbed metabolic pathways involved carnitine shuttle pathway, lipid metabolism, and glucose metabolism. The red color represents the increased level of metabolites, while the blue color represents the decreased level of metabolites. See text for details. PLA2, phospholipase A2; PUFA, polyunsaturated fatty acid; COX, cyclooxygenase; LOX, lipoxygenase; HETE, hydroxyeicosatetraenoic acid; oxoETE, oxo-eicosatetraenoic acid; DTA, docosatetraenoic acid; EPA, eicosapentaenoic acid; HEPE, hydroxyeicosapentaenoic acid; DHA, docosahexaenoic; HDHA, hydroxy docosahexaenoic acid; ALA, α-linolenic acid; CPT1, carnitine palmitoyltransferase 1; CPT2, carnitine palmitoyltransferase 2; TCA cycle, tricarboxylic acid cycle; G-6-P, glucose-6-phosphate; OAA, oxaloacetate; α-KG, α-ketoglutarate; P5C, pyrroline-5-carboxylate

Age-related macular degeneration (AMD) is the leading cause of blindness in old people in developed countries [79]. It was estimated that 288 million people may be affected by AMD in 2040 [80]. The basic pathological characteristic of AMD is the accumulation of drusen that lead to progressive degeneration of photoreceptors and retinal pigment epithelium [81]. On the basis of drusen size, AMD is classified as early AMD and intermediate AMD. These two forms if AMD may progress into the late-stage AMD (or advanced AMD), in the form of neovascular (or “wet”) AMD or as atrophic (or “dry”) AMD [79]. Despite the discovery of anti-VEGF, agent treatment is a milestone in wet AMD treatment, patients’ responses to VEGF inhibitor differ, and the vision is unstable in the long term. As for dry AMD, nowadays treatment is so far from satisfactory. Further understanding the pathogenesis of AMD provides potential targets for treatment [82]. Hence, the metabolomics profiling of AMD may help understand the pathophysiology of AMD, find biomarkers for the prediction and prognosis of AMD, and discover new treatments to achieve advances in 3P medicine (Fig. 3).

AH is an important source of metabolomics in AMD. Dysregulated lipid and glucose energy metabolism was speculated to be the driven force for wet AMD [83]. Hence, researchers conducted a targeted metabolomics study on the glucose metabolism and an untargeted metabolomics study [29, 30]. The prior study analyzed the AH samples of 25 AMD patients and 25 controls via LC–MS [29]. Increased levels of citrate and isocitrate and decreased levels of succinate and α-ketoglutarate were observed in AMD, suggesting dysregulation of glucose metabolism. Moreover, the lower glutamine and higher glutamate levels were detected in AMD. In the latter study, AH samples of 26 AMD patients and 20 controls were analyzed via LC–MS/MS [30]. Eighteen significantly altered metabolites were discovered, including decreased creatine level, amino acid metabolism, carnitine metabolism, and compromised carbohydrate metabolism. These results complemented the map of dysregulated carbohydrate, amino acid, and fatty acid metabolism in wet AMD.

Studies on blood metabolomics have significantly increased recently. Lipid metabolism is believed to be the main disturbed metabolic pathway [84]. Laíns et al. analyzed the serum samples of 89 AMD patients and 30 controls via LC–MS/MS [31]. Eighty-seven metabolites differ in the two groups. Most different metabolites (82.8%) were involved in glycerophospholipid metabolism particularly, supporting the lipid hypothesis in AMD pathogenesis [85]. Importantly, 89 AMD patients were further classified into three groups according to severity stages, and 48 metabolites were found to differ significantly. In a large metabolomics analysis in AMD, Acar et al. analyzed the serum samples of 2267 AMD patients and 4266 controls via ^1^H-NMR [32] and identified 60 metabolites associated with AMD. Increased levels of large and extra-large high-density lipoprotein (HDL) subclasses and decreased levels of very low-density lipoprotein (VLDL) were identified, in agreement with previous evidence [86].

Increased oxidative stress generated by oxidation of lipids also plays a role in the occurrence of AMD [87, 88]. Carnitine is a dipeptide facilitating the transport of fatty acids into the mitochondria to oxidate. Several studies have identified the increased level of long-chain acylcarnitines from the carnitine shuttle pathway in blood samples of AMD patients [33–35], confirming the alteration of carnitine shuttle. These alterations provided evidence for the progressive dysfunction of mitochondrial fatty acid metabolism in AMD. Moreover, several prospective cohort studies proved that moderate intakes of EPA (eicosapentaenoic acid) and docosahexaenoic acid (DHA) were associated with lower AMD incidence rate and less development [89, 90]. In line with these results, Orban et al. identified early molecular markers, DHA and ARA, for retinal degeneration by using MS analyses of mouse models and verified it in serum samples of AMD patients [36]. An untargeted metabolomics study analyzed the plasma samples of 127 wet AMD patients and 50 controls [37]. Seventeen differential metabolites were identified between AMD patients and controls, and most of them were oxidized lipids involved in ARA metabolism.

Furthermore, in a large metabolome association analysis in AMD, 57 of 60 metabolites associated with AMD were associated with complement activation levels [32]. In accordance with the result of genome and proteomics studies, these results further supported the complement activation in AMD [91, 92]. However, how these differential metabolites of AMD interact with the complement system remains unknown. Further investigations of complement activation pathways pave the way for complement inhibition therapies.

In addition to the above cross-sectional studies, a prospective study first evaluated the association between plasma metabolites and progression of AMD via metabolomics [38]. Based on color fundus photos, pentose and glucoronate interconversions pathway and pregnenolone steroids were associated with 3-year AMD progression. Based on dark adaptation (DA) test, glutamine and alanine, aspartate, and glutamate pathways were associated with 3-year AMD progression.

Considering the fact that there are no diagnostic tools to predict patients’ response to anti-VEGF therapy, a metabolomic analyses were conducted to discover predictive metabolites for responsiveness to anti-VEGF therapy [93]. The serum level of glycerophosphocholine, lysoPC (18:2), and PS (18:0/20:4) were higher in non-responders. Biomarkers identifying patients with a poor response to anti-VEGF therapy indicate the possibility of personalized treatment, such as an early switch to different agent or class of drug [94].

In summary, combining these molecular risk factors with previously known biomarkers may optimize the prediction models of AMD progression and lead to preventive strategies and personalized treatments [95].

### Glaucoma

**Fig. 4:**
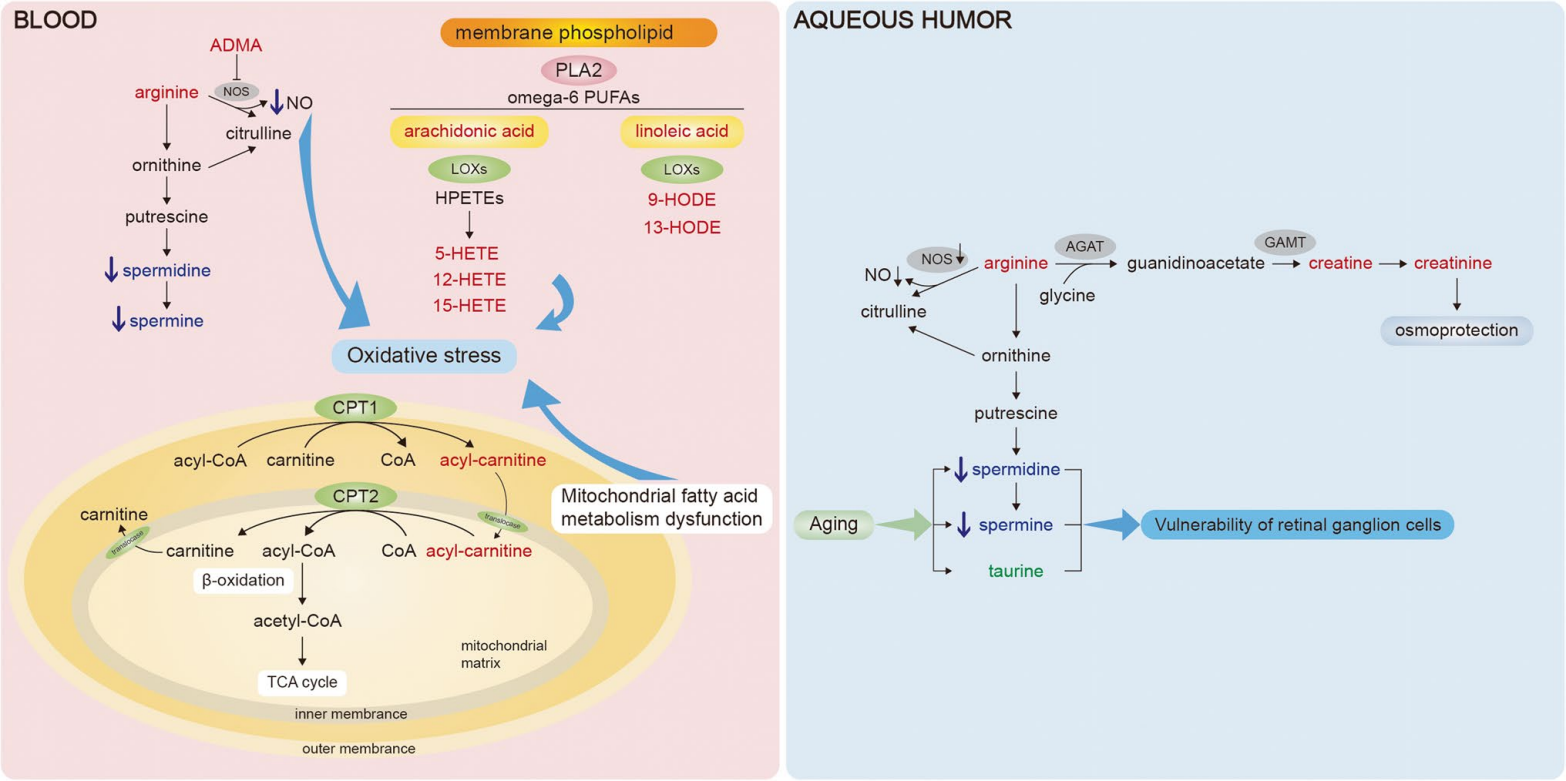
The main disturbed metabolites and metabolic pathways in glaucoma. Blood and aqueous humor samples are collected for metabolomics of glaucoma. The mainly disturbed pathways involved lipid metabolism and carnitine shuttle pathway, and mainly disturbed metabolites involved creatine, spermine, taurine, and others. The red color represents the increased level of metabolites, the blue color represents the decreased level of metabolites, and the green color represents that the level of metabolites is inconsistent. See text for details. ADMA, asymmetric dimethylarginine; NOS, nitric oxide synthase; NO, nitric oxide; PLA2, phospholipase A2; PUFA, polyunsaturated fatty acid; LOX, lipoxygenase; HPETE, hydroperoxyeicosatetraenoic acid; HETE, hydroxyeicosatetraenoic acid; HODE, hydroxy-octadecadienoic acid; CPT1, carnitine palmitoyltransferase 1; CPT2, carnitine palmitoyltransferase 2; TCA cycle, tricarboxylic acid cycle; AGAT, arginine:glycine amidinotransferase; GAMT, guanidinoacetate methyltransferase)

Glaucoma is the most common cause of irreversible blindness worldwide [96]. It was estimated that 111.8 million people may be affected by glaucoma in 2040 [97]. Glaucoma is divided into primary glaucoma, secondary glaucoma, and congenital glaucoma. Primary glaucoma is divided into primary open-angle glaucoma (POAG) and primary angle-closure glaucoma (PACG) according to status of the anterior chamber angle. Many patients are asymptomatic in the early course of disease until central visual acuity is affected in the late stage. Therefore, to prevent blindness from glaucoma, early detection and effective personalized treatment of predisposed individuals are top priorities [62]. Researchers have been dedicated to developing predictive diagnostic approaches [98], including the red blood cell distribution width, short axial length, and circulating blood platelet-to-lymphocyte ratio [99–101]. Moreover, deeper understanding of pathogenesis of glaucoma makes personalized treatment possible [102]. Metabolic profiles of metabolic disorders in glaucoma provide better knowledge of the pathophysiological changes of glaucoma neuropathy (Fig. 4).

Buisset et al. analyzed the AH samples of 26 POAG patients and 26 controls via LC–MS [39]. Twenty-three metabolites allowed discrimination between the two groups. Decreased levels of spermine and taurine were found in POAG. In line with the results of AH, decreased levels of spermidine and spermine were also observed in the plasma of POAG patients [103]. Both spermidine and taurine had a protective effect against neurodegeneration; the decreased level of which in POAG indicated damaged neurons and oxidative stress [104, 105]. However, another study of AH samples of 30 POAG patients and 30 controls via ^1^H-NMR reported increased taurine level in POAG patients [40]. Since the neuroprotective effect of taurine was clear, these divergent results may due to different severities of glaucoma. The author speculated that the increased taurine level could indicate the neuroprotective mechanism against increased oxidative stress. Besides, increased concentration of amino acids, in particular glutamate, was reported. Glutamate, recognized to have excitotoxicity to retinal ganglion cell (RGC), may contribute to glaucomatous neuropathy. Tang et al. analyzed the AH samples of 28 POAG patients and 25 controls and found 22 differentially expressed metabolites [41]. They reported cyclic AMP, 2-methylbenzoic acid, and 3′-sialyllactose in the AH as potential biomarkers for POAG.

Altered lipid metabolism has also been reported in blood samples of glaucoma. Burgess et al. analyzed plasma samples from 72 POAG patients and 72 controls via LC–MS/MS [42]. Increased level of palmitoylcarnitine reflected dysregulated mitochondrial fatty acid metabolism. Furthermore, Umeno et al. emphasized the oxidation products of ARA (HETEs) and linoleates (hydroxylinoleates (HODE)) in POAG, the most abundant PUFAs in vivo [43]. Elevated levels of total HETEs and HODEs manifested the oxidation and inflammation state of POAG.

NO pathway and endothelial dysfunction are also involved. Javadiyan et al. analyzed the serum samples of 211 glaucoma patients and 295 controls via LC–MS/MS, targeting ADMA, symmetric dimethylarginine (SDMA), and L-arginine levels [44]. A significant increase in both serum ADMA and SDMA concentration was detected in advanced glaucoma, supporting the association between increased level of ADMA and SDMA and glaucoma severity [106, 107]. These results further support the potential of NO as a drug target for glaucoma [108], which was applied in a recent clinical trial [109].

Nzoughet et al. analyzed the plasma samples from 34 POAG patients and 30 controls via LC–MS [45]. Nine metabolites were identified, and the most prominent alterations were nicotinamide, N-acetyl-L-leucine, and arginine concentrations. Retinal level of nicotinamide adenine dinucleotide (NAD +) decreases with age, which is common in neurodegenerative disease. It is worth mentioning that oral administration of the NAD + precursor nicotinamide prevented glaucoma in DBA/2 J mice by modulating mitochondrial vulnerability [110].

The population-based studies have been used to investigate the metabolites level in the general population. To figure out the relationship between levels of circulating metabolites and IOP [111], both metabolomic and genetic data were analyzed, and it showed that genetic factors raising O-methylascorbate levels were associated with lower IOP. Besides, lower serum levels of vitamin C were detected in normal-tension glaucoma patients. These studies demonstrated the underlying association between vitamin C metabolism and IOP. A recent study investigated the associations between the level of lipid-related metabolites in blood and POAG [112]. The study showed that higher blood level of total HDL3 cholesterol was significantly associated with lower odds of POAG, and there was a significant causal association between HDL3 cholesterol and POAG. This study provided evidences for the role of dysregulation of cholesterol transport in the pathogenesis of POAG.

In fact, glaucoma is a kind of neurodegenerative disease. Dysregulated glucose metabolism, lipid metabolism, and other metabolic disorders are believed to play a role in neurodegenerative diseases, and therapies targeting metabolic impairments have been proposed [113, 114]. More studies are needed to verify the similarities and discover unique dysregulated pathways in glaucoma and to find new therapeutic opportunity.

### Dry eye disease

Dry eye disease (DED) is characterized by loss of homeostasis of the tear film, such as tear film instability, hyperosmolarity, and ocular surface inflammation and damage [115]. Due to the heterogeneous symptoms, the diagnosis and prognosis of DED are far from satisfactory [116]. As a result, biomarkers have been identified to improve the recognition, stratification, and treatment of patients with DED for 3P medicine practice [117]. However, the application of metabolomics in DED remains limited and is summarized by Yazdani et al. in 2019 [118].

Considering the hypothesis of sex hormone imbalance in DED, Pieragostino et al. analyzed steroid levels in tear samples of 14 DED female patients and 13 female controls via LC–MS/MS [46]. Lower levels of cortisol, 4-androstene-3,17-dione (ADIONE), and 17α-hydroxyprogesterone (17-OHP) in DED patients were observed, consistent with the results of serum [119]. The combination of these three steroids demonstrated the good diagnostic power. A population-based cross-sectional study also reported decreased serum androgens to be highly associated with DED [119]. Androgen deficiency may participate in the pathological processes in DED and act as a biomarker [120]. Most clinical trials supported the therapeutic potential of androgen, with alleviated dry eye-related symptoms and increased tear secretion [121].

### Myopia

Myopia refers to the images of distant objects coming into focus in front of the retina, resulting in blurred distance vision [122]. It was estimated that 4758 million people worldwide had myopia, and 938 million people had high myopia in 2050 [123]. Uncorrected myopia may lead to vision impairment. Since patients with high myopia are more likely to suffer glaucoma, cataract, and other serious ocular diseases, efforts have been made to find potential biomarkers for predicting high myopia.

Metabolomics of AH has been used to identify the metabolic phenotype of high myopia [47]. Twenty-one significantly different metabolites and lipid signatures distinguished patients based on the severity of myopia. The representative abundant metabolites in high myopia were aminooctanoic acid, arginine, and citrulline, while aminoundecanoic acid and cysteinylglycine disulfide were less abundant in high myopia [47].

In addition to AH samples, serum samples were also analyzed. Kearney explored the association between myopia and serum concentrations of dopamine and melatonin in human [48]. Myopes demonstrated a three-time greater melatonin concentration than non-myopes. Similarly, nine dysregulated pathways were observed in myopia, and seven of them were related with oxidative stress and dopamine receptor D2 [124]. Dai et al. analyzed serum samples of 30 high myopia patients and 30 controls via LC–MS [49]. Nine metabolites were identified to be closely correlated with myopia, and eight of them were identified in validation set, indicating disturbed phospholipid catabolism, diacylglycerol metabolism, amino acid metabolism, and vitamin metabolism. Abnormal metabolic pathways represented oxidative stress and inflammation in high myopia. Moreover, the study indicated γ-glutamyltyrosine and 12-oxo-20-trihydroxy-leukotriene B4 as potential biomarkers for high myopia.

### Rhegmatogenous retinal detachment

Rhegmatogenous retinal detachment occurs when liquefied vitreous humor penetrates under the retina through a retinal tear, manifested as a separation of the neurosensory retina from the retinal pigment epithelium [125]. RRD is an ophthalmologic emergency which leads to vision loss without treatment on-time. Proliferative vitreoretinopathy (PVR) is the major complication of RRD [126]. Surgery is the only effective treatment of RRD until now. Metabolomics may help to explore potential therapies.

Li et al. analyzed the vitreous fluid from 8 RRD patients, 9 PVR patients, and 6 controls via LC–MS [50]. Thirty-one metabolites were identified between RRD or PVR patients and controls, and most of them were related to inflammation, proliferation, and energy dysfunction. Twenty-six biomarkers were in the same trends between RRD and PVR groups, while five biomarkers were in different trends. This reflected underlying differences between RRD and PVR. Moreover, histidine metabolism and TCA cycle were seriously disturbed during the formation of RRD and PVR, indicating dysfunction of the energy metabolism.

### Keratoconus (KC)

Keratoconus (KC) is progressive thinning and ectasia of the cornea, leading to myopia, irregular astigmatism, and vision impairment [127]. Environmental and genetic factors may contribute to its development. Potential biomarkers identified by metabolomics provide better understanding of its pathophysiology.

Karamichos et al. analyzed the tear samples of 16 KC patients wearing lenses, 14 KC patients with no correction, and 15 controls via LC–MS/MS [51]. Dysregulated metabolic pathways included glycolysis, gluconeogenesis, TCA, urea cycle, and oxidation state (redox) metabolism. DaphneTeh et al. analyzed the serum samples of 20 KC patients and 20 controls via LC–MS [52]. The analysis revealed that metabolites from the steroidal hormone synthesis pathway (dehydroepiandrosterone sulfate (DHEA-S)) and the ARA pathway (eicosanoids) were upregulated in keratoconus patients, while the levels of PS were downregulated. The increased level of DHEA-S was consistent with the result of saliva samples of KC patients [128], indicating the role of hormones in keratoconus. These analyses further indicated the existence of oxidative stress and inflammation in the pathophysiology of KC [129].

### Other ocular diseases

Despite the previous mentioned ocular diseases, metabolomics is applied in several other diseases. Uveitis is a series of diseases characterized by inflammation inside the eye, caused by inflammation or disordered immune response [130]. Young et al. analyzed the vitreous samples of 20 chronic non-infectious uveitis, 9 lens-induced uveitis, and 13 other ocular diseases via ^1^H-NMR [131]. Increased levels of oxaloacetic acid, glucose, and urea in vitreous humor were observed in lens-induced uveitis as compared with chronic non-infectious uveitis.

Cataract was the first cause of blindness in those aged 50 years and older and caused 0.86 million cases of blindness in 2020 [57]. Tsentalovich et al. and Yanshole et al. analyzed the lens and AH samples of normal aged and cataractous patients via ^1^H-NMR and LC–MS [53, 54]. They observed reduced levels of antioxidants, UV filters, and osmolytes in the cataractous lenses, indicating that the age-related nuclear cataract development may originate from the dysfunction of the lens epithelial cells.

Macular telangiectasia type 2 (MacTel) is a bilateral disease of unknown causes. A genome-wide association study revealed that the glycine-serine metabolism genes were relevant to the disease [132]. Bonelli et al. verified previous genetic findings through metabolomics profiling of serum samples from 60 MacTel patients and 58 controls, with decreased levels of glycine and serine [55]. Furthermore, 73% of the significantly changed metabolites were lipids, including increased phosphatidylethanolamines, lysophosphatidylethanolamines and diacylglycerols, and decreased sphingomyelins. Alterations in these lipid groups had no connection with known risk factors, indicating other unidentified risk factors.

## Future perspectives

Despite the fact that metabolomics has achieved great progression in recent years, many significant challenges remain to be settled [133]. The first is the low number of annotated metabolites. Because only 15–30% of the detected spectra can be identifiable and quantifiable, there remains a large number of un-annotated metabolites [134]. The Human Metabolome Database (HMDB) [135] and METLIN database [136] are the most often used databases in metabolomics. Since the database has been established, it keeps extending the number of metabolite entries to solve this problem. The improvement of database helps to widen our horizon. The second is the variability in metabolite identifications in the same features. Incomprehensive, unstable, and changeable metabolite spectrums are considered common features of metabolic disturbance. Besides, metabolic pathways in human body are complicated, and one metabolite may participate in several pathways. Thus, it is an extremely challenge to determine specific pathways are impacted by the altered metabolites. Concerning these limitations, researchers have been working hard to seek for novel solutions [133].

In regard to the experimental design, because of the differences in sample collections and data analyses, some results lack comparability. There is an urgent need for the standardized protocols of metabolomics analyses. In fact, many studies lack the validation cohort to verify the validity of the potential biomarkers. Moreover, the cross-sectional association studies cannot infer causality, so a large scale of prospective longitudinal studies is needed to establish the metabolic spectrum of the disease. Confounding factors, including diet, gender, systemic and underlying diseases, and known influencing factors, should also be eliminated. It is worth mentioning that patients often have concomitant systemic diseases because multiple risk factors have systemic influences. Patients involved in clinical studies may have other metabolic disorders to different extent. As a result, the varying percentage distribution and different clinical stages of these systemic diseases alone may have a significant impact on metabolomics results. Although a disease-specific metabolomics combination of different factors is hard, when analyzing the baseline data, scientists should make sure that the clinical parameters were comparable, such as blood pressure, blood cholesterol levels, and disease duration.

Last but not least, application of metabolomics in ophthalmology has an unavoidable restriction. The samples specific to ocular diseases, such as vitreous fluid and aqueous humor, are more difficult to obtain and small in volume, placing greater demands for the sensitivity of metabolomics technology.

It is worth mentioning that the combination of metabolomics and other omics has been used in many diseases [137–139]. It is believed that the metabolome affects cellular physiology through modulation of other omics, including the genome, epigenome, transcriptome, and proteome [2]. Multi-omic integration will initiate an additional layer of information to improve and verify the understanding of metabolic processes [2].

## Conclusions and expert recommendations in the context of 3P medicine

Metabolomics is an emerging approach to untargeted screen the metabolic changes of biofluids, cells, and tissues in physiological or pathophysiological processes [140]. In the field of ophthalmology, the discovery of biomarkers by metabolomics improves the prediction and early diagnosis of ocular diseases, promotes the development of targeted prevention in primary care, and enables personalized treatments.

First, all above metabolomic analyses revealed altered metabolites and some of the metabolites exhibiting significant predictive associations with ocular diseases. Biomarkers for disease incidence and progression can serve as indication for intensive risk factor control and early treatment [141]. Although these metabolites provide a predictive medical approach for severe blindness diseases, larger and standardized clinical studies are needed to change clinical practice.

Second, some of these potential biomarkers have preventive potentials. Some metabolites were identified as protective metabolites for DR by in vitro and in vivo study, such as PGF2α and creatine [27, 72]. In fact, most studies only revealed disturbed metabolites in diseases and do not further explore the preventive effects in secondary care. As a result, further experimental and clinical assessment of these biomarkers is needed to find the most effective targeted prevention of ocular diseases.

Third, the prediction of individuals’ response to therapies enables doctors to choose a more suitable and cost-efficient treatment, improving patients’ prognosis and saving medical resources.

Last but not least, metabolomics analysis on a larger population provides evidence for health screening. By combing the molecular biomarkers with current biomarkers, we can better distinguish the population with high risks and make targeted approaches in the early stage to prevent or delay the disease.

These benefits given by metabolomics constitute the main pillars of 3P medicine strategies. To sum up, in primary care, biomarkers prompt population screening and targeted preventive approaches; in secondary care, the discovery of biomarkers enables the early diagnosis of diseases, and the promotion in disease stratification enables personalized treatments; in tertiary care, biomarkers predict patients responses to the therapy and help the doctors to make a cost-effective decision. In this way, current metabolomic studies can benefit both patients and healthcare systems and promote the paradigm shift from reactive to 3P medicine.

Despite the great potential of metabolomics in 3P medicine, most of the biomarkers are not applied to clinical practice. Efforts are needed to accelerate the implementation of biomarkers in 3P medicine. In the field of ophthalmology, the implementation of 3P medicine not only benefit patients suffering from vision loss, increasing their life quality, but also benefit the public via population screening project. For further studies, standardized protocols of metabolomics analyses and a large scale of prospective study combined with other biomarkers are needed to clarify the practicability of molecular biomarkers in the context of 3P medicine.

In summary, metabolomics establishes the metabolic phenotypes of an individual, reflecting the comprehensive outcomes of environment, genes, mRNA, and proteins. The exploration of disease metabolic spectrum has enriched the understanding of the pathophysiology of ocular diseases, thus enabling predictive diagnostics, targeted prevention, and personalization of medical services to the patient. In this review, we give a brief description of the procedure of metabolomics and summarize the relationship between altered metabolic pathways and ocular diseases based on current metabolomics studies in the framework of 3P medicine. Considering the fact that global governments are emphasizing the primary and secondary care in the management of diseases, we believe in the potential of metabolomics in the paradigm shift from reactive to 3P medicine.

## Data Availability

Not applicable
